# Recent advances in neuropeptide-related omics and gene editing: Spotlight on NPY and somatostatin and their roles in growth and food intake of fish

**DOI:** 10.3389/fendo.2022.1023842

**Published:** 2022-10-04

**Authors:** Xiaozheng Yu, Haijun Yan, Wensheng Li

**Affiliations:** State Key Laboratory of Biocontrol, Guangdong Province Key Laboratory for Aquatic Economic Animals, Provincial Engineering Technology Research Center for Healthy Breeding of Important Economic Fish, Institute of Aquatic Economic Animals, School of Life Sciences, Sun Yat-Sen University, Guangzhou, China

**Keywords:** neuropeptidomics, gene editing, neuropeptide Y, somatostatin, feeding, growth

## Abstract

Feeding and growth are two closely related and important physiological processes in living organisms. Studies in mammals have provided us with a series of characterizations of neuropeptides and their receptors as well as their roles in appetite control and growth. The central nervous system, especially the hypothalamus, plays an important role in the regulation of appetite. Based on their role in the regulation of feeding, neuropeptides can be classified as orexigenic peptide and anorexigenic peptide. To date, the regulation mechanism of neuropeptide on feeding and growth has been explored mainly from mammalian models, however, as a lower and diverse vertebrate, little is known in fish regarding the knowledge of regulatory roles of neuropeptides and their receptors. In recent years, the development of omics and gene editing technology has accelerated the speed and depth of research on neuropeptides and their receptors. These powerful techniques and tools allow a more precise and comprehensive perspective to explore the functional mechanisms of neuropeptides. This paper reviews the recent advance of omics and gene editing technologies in neuropeptides and receptors and their progresses in the regulation of feeding and growth of fish. The purpose of this review is to contribute to a comparative understanding of the functional mechanisms of neuropeptides in non-mammalians, especially fish.

## 1 Introduction

In recent ten years, the rapid development of omics technology has introduced us to the era of big data. The omics technology consists of four major groups, namely genomics, transcriptomics, proteomics and metabolomics (1). Genomics studies the genetic structure composition of biological systems (2); transcriptomics is about gene transcription in cells and the regulation of transcription at the overall level (3); proteomics investigates the proteins expressed by biological systems and their differences caused by external stimuli (4); and metabolomics examines the changes of metabolites produced by organisms under different conditions (5). Just as in the functional study of other target genes, the application of omics technology in the functional study of neuropeptides and their receptors also provides a large amount of data, opening up new directions for research in this field.

The establishment of gene editing technology is a landmark event in the life sciences. The success of specifically targeting DNA at any given location within the genome has enabled many interesting possibilities that have inspired scientists for decades. A variety of methods have been used in genetic engineering to modify DNA sequences in the genome. Gene targeting and gene trapping are two techniques for constructing knockout animals from embryonic stem cells (6). Gene targeting replaces the endogenous gene by homologous recombination to knock out the target gene, while gene trapping has two methods, promoter trapping and polyA trapping (7, 8). With the continuous development of life science, researchers have successively developed zinc finger nucleases (ZFNs) (9), transcription activator-like effector nucleases (TALENs) (10), and clustered regularly spaced short palindromes Repeat-CRISPR-associated 9 (CRISPR/Cas9) (11), which have provided a broad range of efficient tools for life and medical research.

Omics and gene editing approaches have been employed in the study of the mechanisms by which neuropeptides and their receptors regulate feeding and growth. For vertebrates, the ability to regulate food intake based on the energy storage status of the body is essential for growth and even survival. The physiological mechanisms controlling feeding and growth are relatively conserved in vertebrates, and many of the neuropeptides that centrally regulate feeding and growth in mammals have been identified successively in fish (12, 13). Fish are an extremely diverse group of species with great differences in their ecological niches, habitats, life histories and feeding behaviors at different life stages (14). In fish, many neuropeptides involved in feeding regulation have been isolated or their protein sequences have been deduced by cloning their cDNA sequences. Neuropeptide Y (NPY), isolated firstly from porcine brain, is a strong orexigenic neuropeptide in the hypothalamus (15), and other feeding-promoting neuropeptides such as orexin and ghrelin have also been identified in fish (16, 17). Somatostatin (SS) has been firstly known to inhibit the secretion of pituitary growth hormone (GH) and is later found to have feeding inhibitory effects as well (18). Other feeding-inhibiting neuropeptides, proopiomelanocortin (POMC) (19), corticotropin-releasing factor (CRF) (20), cholecystokinin (CCK) (21), and etc., have also been identified in fish. These neuropeptides act as feeding regulators by binding to the corresponding receptors and activating downstream signaling pathways, and the interactions between these neuropeptides have also been shown to regulate feeding in fish (22, 23).

Our objectives are firstly, to review the application and development of omics and gene editing technologies in the field of functional studies of neuropeptides and their receptors; secondly, to summarize the research progress of neuropeptides involved in fish feeding and growth regulation, focusing on their different types, distribution patterns and functional effects mediated by different receptors.

## 2 Neuropeptidomics and gene editing

### 2.1 Neuropeptidomics related to neuropeptides and relevant receptors

Neuropeptide research has been greatly promoted in recent years, due to the rapid emergence of omics technologies. Neuropeptides are mainly dependent on the corresponding receptors for executing their function. Therefore, the next section will explore the recent advances in the omics of neuropeptide and its receptors (**Table 1** for detail).

**Table 1:** Recent advances in the omics of neuropeptide and its receptors.

Omics	Species	Physiological functions	References
Neuropeptides	NPY		
Transcriptome	Mice (*Mus musculus*)	Food intake and stress	(24–26)
	Giant grouper (*Epinephelus lanceolatus*)	Appetite and digestion	(27)
	Chicks (*Pullus*)	Food intake	(28)
	orange-spotted grouper (*Epinephelus coioides*)	Food intake	(29)
	juvenile yellow catfish (*Tachysurus fulvidraco*)	Feeding and growth	(30)
	Kuruma Prawn (*Marsupenaeus japonicus*)	Reproduction	(31)
	Brahman heifers (*Bos indicus*)	Reproduction	(32)
Proteomics	Mice (*Mus musculus*)	Neurotransmitter secretion, neurodegenerative disorder	(33–35)
	Pig (*Susscrofa domestica*)	Neuroinflammation	(36)
metabolomics	Human (*Homo sapiens*)	Tumors growth	(37)
**Neuropeptides**	**POMC**		
Transcriptome	Mice (*Mus musculus*)	Food intake	(38)
	Siberian hamster (*Phodopus sungorus*)	Seasonal plasticity in energy balance	(39)
	zebra finch (*Taeniopygia guttata*)	Parental care	(40)
	Goat (*Capra hircus*)	Analgesia	(41)
	Blackheaded buntings (*Emberiza melanocephala*)	Seasonal life-history	(42)
	Human (*Homo sapiens*)	Hair follicle regeneration, insulin sensitivity	(43, 44)
	*Takifugu rubripes*	Hypoxia stress	(45)
Proteomics	Rat (*Sprague-Dawley*)	Stress	(42)
	Mice (*Mus musculus*)	Insulin response	(46)
single-cell transcriptomics	Mice (*Mus musculus*)	Development	(47, 48)
**Neuropeptides**	**CCK**		
Transcriptome	Mice (*Mus musculus*)	burn-induced pain, food intake,	(49, 50)
	Fruit fly (*Drosophila melanogaster)*	Social isolation	(51)
	Nile tilapia (*Oreochromis niloticus*)	Reproduction	(52)
	*Pelteobagrus vachellii*	Food intake	(53)
	orange-spotted grouper (*Epinephelus coioides*)	Food intake	(29)
Proteomics	Mice (*Mus musculus*)	Food intake	(54)
	Cell line (from mice, *Mus musculus*)	Digestion	(55)
metabolomics	Mice (*Mus musculus*)	Cold stress	(56)
**Neuropeptides**	**SS**		
Transcriptome	Mice (*Mus musculus*)	Food intake, Depression, Nerve excitation	(57–59)
	Human (*Homo sapiens*)	Bipolar disorder, Pituitary adenomas	(60, 61)
	Spotted scat (*Scatophagus Argus*)	Growth	(62)
	Channel Catfish (*Ictalurus punctatus*)	Vaccines to protect fish	(63)
	Yellow catfish (*Tachysurus fulvidraco*)	Environmental influences on feeding	(30)
	Nile tilapia (*Oreochromis niloticus*)	Salinity affects growth	(64)
	*Coilia nasus*	Food intake	(65)
	Zebrafish (*Danio rerio*)	Glucose metabolism	(66)
Proteomics	silver carp (*Hypophthalmichthys molitrix*)	Hypoxia	(67)
	Mice (*Mus musculus*)	Alzheimer’s disease, enteropancreatic neuroendocrine tumors	(35, 68)
metabolomics	Mice (*Mus musculus*)	Ischemia-induced retinal cell death, Atractylodis Rhizoma	(69, 70)
**Neuropeptides**	**CRF**		
Transcriptome	Mice (*Mus musculus*)	Learning Behavior, Neurotransmission,	(71, 72)
	Human (*Homo sapiens*)	Pregnancy	(73)
	Atlantic salmon (*Salmo salar*)	Feeding and Stress	(74)
	Pig (*Susscrofa domestica*)	Ammonia poisoning	(75)
Proteomics	silver carp (*Hypophthalmichthys molitrix*)	Hypoxia	(67)
	Human (*Homo sapiens*)	Dry eye syndrome	(76)
metabolomics	Human (*Homo sapiens*)	Air Pollution	(77)
	Pig (*Susscrofa domestica*)	Reproduction	(78)
**Neuropeptides**	**OREXIN A**		
Transcriptome	Pig (*Susscrofa domestica*) Mongolian sheep (*Ovis aries*)	Reproduction Reproduction	(79, 80) (81)
	Pinto abalone (*Haliotis kamtschatkana*)	Feeding and digestion	(82)

#### 2.1.1 Transcriptomics

Since the 1990s, transcriptomics technology has entered a period of vigorous development and has undergone Generation 1, 2 and 3 sequencing technologies, which have been widely used in biological, medical and clinical research as well as drug development (83). This technology has also been widely used in the study of neuropeptides, especially playing an important role in exploring the functions and mechanisms. Neuropeptides have been shown to play an important role in the regulation of physiological activities (84). The introduction of transcriptomics has helped the mechanistic exploration of neuropeptides at the RNA level and the network of interactions. NPY, SS, and CCK have been found to influence feeding behavior, and in recent years new evidence has been found in mammalian (24), avian (85), and fish species (27, 29, 53, 65) using transcriptomics. Among them, NPY is involved in promoting feeding, and analysis of the hypothalamus transcriptome of mice subjected to a daily 2-h heat treatment (36°C) for 14 d suggested that up-regulated neuropeptide Y acted to attenuate reduced food intake (24). It has been reported that in broiler chickens, dietary stevioside (STE) supplementation promoted feed intake, and the analysis of RNA-Seq in the hypothalamus of the STE-supplemented group compared with the control group showed that several appetite-related genes, such as NPY and NPY5R, were differentially expressed, and it was suggested that dietary STE supplementation promoted feed intake through the regulation of the hypothalamic neuroactive ligand-receptor interaction pathway and the alteration of intestinal microbiota composition (85). On the contrary, SS is involved in suppressing feeding, especially important in studying the mechanism of feed additives (28) and environmental influences on feeding (30). In yellow catfish (*Tachysurus fulvidraco*), it has been reported that sertraline (SER) increased shoaling, decreased food consumption, higher cannibalism rate and RNA-seq results showed that transcript levels of SS reduced in the brain following SER exposure, indicating that SER disturbs neuropeptides which may be unrelated to its antidepressant effects in vertebrates (30). Reproductive activity is endocrinologically regulated by many peptide hormones, and a more detailed transcriptome of the process of reproduction has been made in mammals (79, 80). Orexin is involved in the regulation of the reproductive system through the hypothalamic-pituitary-ovarian axis (79), and the transcriptome profile of the endometrium during early pregnancy in pigs has revealed that orexin affected the expression of a large number of genes differentially (79) while transcriptome of myometrial explants during the early implantation period suggested that orexin influenced the process involved in quiescence, proliferartion, remodeling and angiogenesis in myometrial explants during the peri-implantation period in the pig (80). NPY has also been found to be involved in gonadal development in crustaceans (31) and mammals (32). CCK receptor (CCKAR and CCKBR) deletion leads to abnormal cortical development, and using comparative transcriptomic analysis, transcripts of CXCL12, BMP7 are downregulated and may be target genes for abnormal cortical development (86). Neuropeptides are also involved in mood management, stress regulation. Transcriptome analysis of somatostatin interneurons in nucleus accumbens after cocaine exposure identified JUND transcription factor as a key regulator of cocaine (57) and CRF2 is also involved in cocaine-induced neuroexcitation (71). Somatostatin interneurons are involved in emotion regulation, and transcriptome analysis of somatostatin interneurons in male and female mice under chronic stress conditions separately revealed that chronic stress leads to gene dysregulation in several key pathways, including sex-specific differences in somatostatin interneuron profiles (87). These changes occurred mainly in the reduced expression of elongation initiation factor 2 (EIF2) signaling, suggesting that dysfunction of the translational machinery of somatostatin interneurons may be critical for the development of depressive behavior in males (87). In addition, somatostatin receptor (SSTR2) is also involved in neuro-emotional regulation (60). And, SSTR4 has a modulatory role in Alzheimer’s disease (88). CCK is involved in nociceptive perception, as the CCK2 receptor (CCKbR) was found to be a pain target in burn-induced nociception and is involved in reducing the effectiveness of opioids (49).

#### 2.1.2 Proteomics

A wealth of functional genomic technologies has emerged as the focus of research has shifted to the gene specific function and understanding the regulation of each identified gene product. The goal of these efforts is to better understand how the information stored in the genome encodes all of the complexity required to sustain complex multicellular organisms (89). Despite the impressive advances made by these technologies, the interpretation of their results is limited without corresponding data on proteins (90). Neuropeptides have also been studied using proteomics and showing that they play an important role in the nervous system. In Parkinson’s disease, a disease of aging associated with vesicular transport dysfunction and neurotransmitter secretion, proteomic sequencing of secretory granules revealed reduced levels of NPY (33). In studies related to depression, NPY and NPY2R protein expression was downregulated in hippocampal proteins of mice in a model of social failure versus control mice (34). SST has been reported to be associated with Alzheimer’s disease, and identified by LC-MS/MS analysis, somatostatin-related amyloidosis is a novel restricted human amyloid type associated with somatostatin -producing enteropancreatic neuroendocrine tumors (36). LC-MS analysis using SST peptide (SST-scFv8D3) injected into the hippocampus of mice revealed the elevated degradation of NPY (35). Using proteomic analysis in renal failure studies, NPY-NPY2R system was found to predict nephrotoxicity and pathogenic effects in the glomerulus (91). Based on the brain proteomic analysis in silver carp (*Hypophthalmichthys molitrix*), it was revealed that hypoxia affected significantly the biochemical indices and neurotransmitter contents in the brain, and SST1A was upregulated as well (67).

#### 2.1.3 Metabolomics

Metabolites represent both downstream outputs from the genome and upstream ones from the environment. Thus, the study of metabolomics allows scientists to explore the relationships of gene-environment interactions. In comparison with genetic and genetic risk scores that can be used to indicate what is likely to happen, metabolic analysis and metabolic phenotyping indicate what is currently happening and what has already happened. In this regard, metabolomics is able to take a look at not only endogenous metabolites (gene-derived metabolites) and exogenous metabolites (environmentally derived metabolites) in the form of disease biomarkers, but also provides unique insights into the underlying causes of disease (92, 93). Retinal ischemia mouse cells treated with neuropeptide PACAP and the somatostatin analog octreotide and tested for anti-ischemic ability, metabolome results show reduced intravascular epidermal growth factor overexpression and glutamate release (69). Using metabolomics analysis, when NPY receptor Y2R activated by NPY, glycolysis is observed to increase while intracellular nutrients are depleted, which may be due to high rates of conversion of glucose to lactate, glycine and alanine. When the Y2R activation is blocked by BIIE0246, the metabolic responses reversed (37). To date, relatively few studies have been conducted on neuropeptides in metabolomics, and more studies are yet to be explored.

### 2.2 Gene editing regarding to ligands and related receptors

Genetic engineering has led to a better understanding of the genetic functions of neuropeptides and related receptors. Gene knockout technology is an irreplaceable part of transgenic technology, and conditional gene knockout technology, as a new generation gene knockout technology, has obvious advantages and wider application compared with the first-generation gene knockout technology (94). Next, advances in the study of neuropeptides and their receptors function using gene editing will be presented (**Table 2** for detail).

**Table 2:** Gene editing applied to the study of neuropeptide and its receptor function.

Species & Gene Edited Animals	Gene editing technology	Physiological functions	References
Neuropeptides	NPY		
Z: (NPY-KO) zebrafish	crispr/cas9	Emotional behaviors	(95, 96)
M:Male NPY knockout mice	Gene targeting (homologous recombination)	kidney disease	(91)
M: Y1R knockout mice	Gene targeting (homologous recombination)	Bone matrix	(97)
M: Npy1r^rfb^ mice	Gene targeting (homologous recombination), Cre/loxP system	Metabolic and behavioral dimorphism	(98)
M: (npy1r-/-), (npy2r-/-) mice	Cre-loxp system	Taste responses	(99)
M: Male NPY knockout mice	Cre-loxp system	Bone metabolism	(100)
M: IR^lox/lox^;NPY^cre/+^ mice	Cre-loxp system	Cognitive function	(101)
M: IR^lox/lox^; NPY^cre/+^ mice	Cre-loxp system	Cognitive Functioning	(102)
M: Agrp ^cre/+^; NPY ^lox/lox^ mice	Cre-loxp system	Food intake	(103)
M: Male NPY knockout mice	Cre-loxp system	Rapid feeding and glucose metabolism	(104)
M: Npy1r^rfb^ mice,	Gene targeting (homologous recombination), Cre/loxP system	Obesity	(105)
M: NPY knockout mice	Cre-loxp system	Skeleton and adiposity	(106)
M: NPY^cre/+^; RANK^lox/lox^ mice	Cre-loxp system	Bone loss	(107)
M: NPY^cre/+^; RANK^lox/+^mice	Cre-loxp system	Bone mass	(108)
M: NPY^creERT2/+^; Lepr^lox/lox^ mice	Cre-loxp system	Energy homeostatic	(109)
M: Y1^lox/lox^; INS2^cre/+^ mice	Cre-loxp system	Glucose metabolism	(110)
R: NPY knockout rats	ZFN	Myocardial infarction	(111)
**Neuropeptides**	**OREXIN**			
M: orexin knockout mice	Gene targeting (homologous recombination)	Sleep regulation	(112)
M: ORX-/-; ORX-tTA mice;ORX-/-; ORX-tTA;TetO-GCaMP6 mice	Gene targeting (homologous recombination)	Cataplexy	(113)
M: Orexin knockout mice	Gene targeting (homologous recombination)	Arousal and reward circuits	(114)
M: Orexin knockout mice	Gene targeting (homologous recombination)	Social fear	(115)
M: Ox1r-deficient mice, Ox2r-deficient mice	Gene targeting (homologous recombination)	Long-term energy metabolism	(116)
M: OX1r knockout mice, OX2r knockout mice	crispr/cas9	Sleep/wake states	(117)
M:Orexin knockout mice	Gene targeting (homologous recombination)	Lipid metabolism	(118)
M: ORX;vGT2-KO mice, ORX-KO mice	Gene targeting (homologous recombination), Cre/loxP system	Body temperature and heart rate	(119)
M: Orexin knockout mice	Gene targeting (homologous recombination)	Wake-promoting	(120)
M: Orexin knockout mice	Gene targeting (homologous recombination)	Sleep breathing	(121)
M: Orexin knockout mice	Gene targeting (homologous recombination)	Fear and anxiety	(122)
M: Orexin knockout mice	tet-off system	Narcolepsy and Orexin system function	(123)
M: Orexin knockout mice	Gene targeting (homologous recombination)	Cocaine-related behaviors	(124)
M: Orexin knockout mice, serotonin knockout mice, Orexin and serotonin double knockout mice	Cre/loxP system	REM sleep and cataplexy	(125)
M: Orexin neuron-ablated mice	tet-off system	Hypothermia	(126)
M: melanin-concentrating hormone-Cre::Orexin-KO mice	Gene targeting (homologous recombination)	Narcolepsy	(127)
M: Orexin knockout mice	Gene targeting (homologous recombination)	Rapid eye movement sleep	(128)
M: Hcrtr1^Dbh-CKO^ mice	Gene targeting (homologous recombination), Cre/loxP system	Sleep	(129)
**Neuropeptides**	**POMC**			
M: POMC GHR KO mice, POMC STAT5 KO mice	Cre/loxP system	Hyperphagia	(130)
M: Pomc promoter-driven Gpr17 knockout (PGKO) mice	Cre/loxP system	Metabolism	(131)
M: POMC-specific protein kinase R-like ER kinase (PERK) deficiency mice	Cre/loxP system	Metabolism	(132)
M: pomc neuronal enhancer (nPE1 and nPE2) knockout mice	Gene targeting (homologous recombination)	Alcohol drinking	(133)
M: Neuronal POMC enhancer knockout (nPE-/-) mice with hypothalamic-specific deficiency of POMC mice	Gene targeting (homologous recombination)	Alcohol drinking	(134)
M: nPE1-/-mice	Gene targeting (homologous recombination)	Alcohol drinking	(135)
M: nPE1-/-mice	Gene targeting (homologous recombination)	Pregnancy and lactation	(136)
M: Pomc conditional knockout mice	Cre/loxP system	Painful neuropathy	(137)
M: miR-17-92 KO mice, miR-7-sp mice	Cre/loxP system	Sex-specific diet-induced obesity	(138)
M: HIF2α knockout in POMC neurons mice	Cre/loxP system	Age-associated metabolic disorders	(139)
M: lacking FKBP51 in Pomc-expressing cells mice	Cre/loxP system	Stress response	(140)
M: deletion of rptor in POMC neurons mice	Cre/loxP system	Oxidative metabolism	(141)
M: Knockdown of the Trpv1 gene in ARC POMC neurons mice	Gene targeting (homologous recombination), Cre/loxP system	Food intake	(142)
M: Bbs1 gene deletion POMC neurons mice	Cre/loxP system	Cardiovascular regulation	(143)
M: POMC neuron-specific deletion of nicotinamide mononucleotide adenylyltransferase 2 (Nmnat2) mice	Cre/loxP system	Lipid and glucose metabolism	(144)
M: selective deletion of the Bbs1 gene in POMC neurons mice	Cre/loxP system	Body weight regulation	(145)
M: Rax-CreERT2:Arc Pomc ^loxTB/loxTB^ mice	Cre/loxP system	Metabolism	(146)
M: SOCS3^flox/flox^/POMC-Cre mice	Cre/loxP system	Metabolic and cardio-vascular regulation	(147)
M:POMC-specific AIF-deficient mice	Cre/loxP system	Metabolism	(148)
M: PTP1B^flox/flox^/POMC-Cre mice	Cre/loxP system	Liver lipids and glucose tolerance	(149)
M: POMC-SRC-2-KO mice	Cre/loxP system	Metabolism	(150)
M: Pomc-Cre; Atg7^loxP/loxP^ mice	Cre/loxP system	Energy balance regulation	(151)
**Neuropeptides**	**SS/SST**			
Z: SST1 knockout zebrafish	CRISPR/Cas9	Growth and metabolism	(152)
Z: SST 4 knockout zebrafish	CRISPR/Cas9	Growth and reproduction	(153)
M: SST knockout mice	Gene targeting (homologous recombination)	Mood symptoms	(87)
M: SST 4 receptor knockout mice	Gene targeting (homologous recombination)	Chronic stress, inflammation, hyperalgesia, and airway hyperreactivity	(154)
M: SST receptor subtype 2 knockout mice, ss receptor subtype 4 knockout mice	Gene targeting (homologous recombination)	Stress response and behavioral emotionality	(154–156)
M: SST receptor subtype 2 knockout mice	Gene targeting (homologous recombination)	Emotional and cognitive ageing	(154–156)
M: SST 2 receptor knockout mice	Gene targeting (homologous recombination)	Regulation of growth hormone secretion	(157)
M: (GHR/IGF1R) SST-specific double knockout (KO) mice	Cre/loxP system	Homeostatic control of GH secretion	(58)
**Neuropeptides**	**CRF**			
M: GrC-CRFR1cKO mice	Gene targeting, Cre/loxP system	Cerebellar learning	(158)
M: CRF2 receptor knockout mice	Gene targeting (homologous recombination)	Stress-induced mast cell degranulation and associated disease pathophysiology	(159)
M: CRFR1-Deficient Mice	Gene targeting (homologous recombination)	Cognitive dysfunction and anxiety-like states induced by cocaine	(160)
M: CRF1 receptor knockout mice and CRF2 receptor knockout mice	Gene targeting (homologous recombination)	Anxiety-like behavior	(161)
M: The medial amygdala specific knockdown of CRFr2 mice	Cre/loxP system	Social behavior	(162)
M:CRFR2 knockout mice	Gene targeting (homologous recombination)	Sexually dimorphic metabolic responses	(163)
M: CRFR2 knockout mice	Gene targeting (homologous recombination)	Cognitive dysfunction	(164)
M: CRF1 receptor knockout mice	Gene targeting (homologous recombination)	Pain perception	(165)
M: CRFR2 knockout mice	Gene targeting (homologous recombination)	Social behavior deficits	(166)
**Neuropeptides**	**CCK**			
M: CCK1 receptor KO, CCK2 receptor KO, and CCK1 receptor and CCK2 receptor double KO mice	Gene targeting (homologous recombination)	Anti-inflammatory actions	(167)
M: CCK knockout mice	Gene targeting (homologous recombination)	Lipid transport	(168)
M: CCK knockout mice	Gene targeting (homologous recombination)	Cholesterol crystallization and gallstone formation	(169)
M: CCKAR knockout mice; CCKBR knockout mice; CCKAR and CCKBR double knockout mice	Gene targeting (homologous recombination)	Taste signaling in the peripheral taste organ	(170)
M: CCK 1R knockout mice	Gene targeting (homologous recombination)	Functional coupling of CCKAR and CCKBR	(171)

?, unknown. Z, zebrafish (Danio rerio); M, mouse (Mus musculus); R, rat (Rattus norvegicus).

#### 2.2.1 Whole body gene knockout

Gene targeting technology is usually used to knock out the target gene, and its essence is to replace the endogenous gene through homologous recombination (172). Using the powerful technology, researchers constructed gene knockout models to explore the function of neuropeptides or their corresponding receptor. It has been reported that transgenic mice deleting NPY were used to investigate albuminuric kidney disease (91). And Y1R knockout mice have been reported to explore the role of Y1R in the regulation of bone and energy homeostasis (97). Ablating orexin in mice have been reported in the fields of sleep regulation, social fear, cataplexy and lipid metabolism (112, 113, 115, 118). Orexin receptor-deficient mice have been used to support the unique role of orexin receptors 1 and 2 in long-term energy metabolism (173). It has been reported that mice lacking SST were used to study the effect of SST in depression (87). In addition, mice deficient in the SST receptor, SSTR2, or SSTR4, have been reported to be models for studying behavioral changes induced by chronic stress (154–156). CCK knockout mice were reported to study the role of CCK in the lipid transport, gallbladder contractile and small intestinal motility (168, 169). Mouse with CCKAR knockout or CCKBR knockout or double knockout were used to study the role of CCK receptors in taste, fear, feeding and bone homeostasis (170, 171). Also, corticotropin-releasing hormone knockout (CRH-KO) mice have been reported to be used in the study of inflammatory bowel disease and stress (174, 175). It has been reported that CRF receptor 1 knockout mice were used in the study of cognitive and anxiety-like states and social behavior (160, 166), while CRF receptor 2 knockout mice were used in the study of inflammation, recognition memory and glucose metabolism (163, 164, 176).

CRISPR/Cas9 is a powerful gene editing tool with the advantages of being efficient, easy, fast and cheap (173). The CRISPR/Cas9 system consists of two components, Cas9, a signature nucleic acid endonuclease, and a guide RNA (gRNA) molecule. Cas9 cleaves the target sequence under the guidance of gRNA, resulting in DNA double-strand breaks. The broken double strand is repaired by homologous recombination, non-homologous end joining and other mechanisms, and abnormal bases are inserted or made absent at the break, resulting in code-shifting mutations, and finally the target gene is knocked out (173). It has been reported that the CRISPR/Cas9 system has been used for the construction of knockout models of neuropeptides and their receptors. In mammals, orexin 2 receptor knockout mice generated by CRISPR/Cas9 system were applied to investigate its role in the regulation of sleep/wake states (117). In zebrafish, CRISPR/Cas9 system was used to knock out NPY to study its effect on emotional behaviors (95, 96). Also, SS receptor 1 and 4 were reported to be knocked out using CRISPR/Cas9 to study their roles in growth and reproduction, respectively (152, 153).

#### 2.2.2 Conditional gene knockout

Conditional gene knockout, an upgraded version of gene knockout, is a very powerful technique for studying the function of neuropeptides and their receptors in the nervous system. Particularly, condition-specific knockout mice based on the Cre-loxP system have been favored by many studies because of their good experimental results. Cre (Cre recombinase) is a protein with a relative molecular mass of 38000 Dalton composed of 343 amino acids encoded and expressed by the cre gene in bacteriophage P1 (177). The loxP (locus of x-over, P1) is a 34 bp sequence found from bacteriophage P1, consisting of two 13 bp inverted repeat sequences and an 8 bp spacer sequence in the middle (178). Cre recombinase can specifically recognize loxP sequences on intracellular genes or DNA, and mediate different specific recombination reactions according to the position of loxP sequences and the relationship between loxP sequences (179). Conditional knockout mice are usually generated by breeding the loxp mice in which loxp flanked alleles of interest gene with Cre mice expressing Cre recombinase under the control of a selected promoter that specifically targets the tissue or cell of interest. Tissue- or cell-specific expression promoters determine the location of Cre expression resulting in conditional gene knockout (180). The Cre-loxp system was first used to specifically knock out the DNA polymerase gene in T lymphocytes (181). Some studies have reported that neuropeptides such as NPY, orexin and their receptors have been conditional knockout in mice based on the Cre-lox system. The effect of NPY on feeding was investigated in mice with specific knockout of NPY on AGRP neurons (103). Male mice targeted disruption of the Npy1r gene in limbic areas were used to study the effect of NPY-Y1R system in energy balance and emotional behavior as well as diet-induced obesity (105). Another study applied mice with β-cell specific ablation of the Y1 receptor to investigate the effect of Y1 receptor on the glucose metabolism (110). Also, mice selectively disrupted the orexin receptor in noradrenaline neurons were generated to study the effect of orexin-to-noradrenaline signaling on the sleep behavior (129). Owing to the Cre-loxp system, researchers could perform specific knockout in the specific neurons of their interest. It has been reported that growth hormone receptor (GHR) or IGF-1 receptor (IGF1R) were ablated in SST neurons specifically to investigate whether GHR or IGF1R is required for the homeostatic control of GH secretion (58). And transgenic mice in which the vesicular glutamate transporter 2 gene was disrupted selectively in orexin-producing neurons were used to study the roles of orexin neurons in mediating methamphetamine-induced changes in body temperature and heart rate (119). It is well known that in the neuropeptide and receptor family, there are usually several receptors corresponding to one ligand, neuropeptides, such as NPY and somatostatin (182, 183). Therefore, the Cre-loxP conditional knockout system is very useful and efficient for identifying specific receptor types that mediate ligand function in specific tissues.

## 3 Neuropeptide Y and somatostatin in food intake and growth of fish

### 3.1 Neuropeptide Y family

#### 3.1.1 Peptides and receptors of NPY family

The ligands of the NPY system, also known as NPY family peptides, are highly conserved in vertebrates in terms of their amino acid sequences and protein structures. PP, PYY and NPY are all composed of 36 amino acids with amidated C-terminus, classified into NPY family (184). In lower vertebrates, the discovery of NPY family polypeptides was mainly achieved through gene cloning. Only NPY and PYY have been cloned or isolated from both jawless and jawed cartilaginous fish, however, PP has not been found yet (185, 186). Among tetrapods, the presence of PP is found exclusively in *Latimeria chalumnae* (187). In summary, there are four NPY family peptides in teleost, namely NPYa, NPYb, PYYa and PYYb (**Table 3**). NPY of teleost is mainly expressed in the central nervous system (217, 229). In addition to the CNS, NPY mRNA levels has been detected in some peripheral tissues such as kidney, intestine, eye and pituitary (206, 210, 215). Studies have shown that PYYa of zebrafish (*Danio rerio*) (229), goldfish (197), grass carp (*Ctenopharyngodon idellus*) (193) and tilapia (220) is mainly expressed in the brain and spinal cord, while PYYb of grass carp (194), Japanese Flounder (*Paralichthys olivaceus*) (204), yellowtail (*Seriola quinqueradiata*) (230) and red-bellied piranha (*Pygocentrus nattereri*) (200) is mainly expressed in the foregut and midgut, and also expressed to a certain extent in the brain.

**Table 3:** Neuropeptide Y and its receptors on growth and food intake in fish.

Species	Neuropeptides	Receptors	Physiological functions	Major references
Estuarine tapertail anchovy (*Coilia nasus*)	PYYb		Food intake	(188)
Zebrafish (*Danio rerio*)	NPYa, PYYa, PYYb	Y1,Y2, Y2-2, Y4, Y7, Y8a, Y8b	Food intake	(189–192)
Grass carp (*Ctenopharyngodon idellus*)	NPY, PYYa, PYYb	Y8a, Y8b	Food intake, energy metabolism	(193–195)
Indian Major (*CarpCirrhinus cirrhosus*)	NPY	Y2	Regulation of LH secretion	(196)
Goldfish (*Carassius auratus*)	NPY, PYYa		Food intake	(197, 198)
Ya fish (*Schizothorax prenanti*)	NPY		Late embryonic development, food intake	(199)
Red-bellied piranha (*Pygocentrus nattereri*)	PYY		Food intake	(200)
Cavefish (*Astyanax fasciatus mexicanus*)	PYYb		Food intake	(201)
Glass catfish (*Kryptopterus vitreolus*)	NPY		Food intake	(202)
Yellow catfish (*Pelteobagrus fulvidraco*)	NPY		Food intake	(203)
Yellowtail (*Seriola quinqueradiata*)	NPY		Digestion, food intake	(204, 205)
Atlantic salmon (*Salmo salar*)	NPY, PYYa		?	(13, 206)
rainbow trout (*Oncorhynchus mykiss*)	NPY, PYY	Y2, Y2-2, Y4, Y7, Y8a, Y8b	Food intake, fatty acid sensing and metabolism.	(207–209)
Atlantic cod (*Gadus morhua*)	NPY	Y8b	Food intake, growth	(210–212)
Winter flounder (*Pseudopleuronectes americanus*)	NPY		?	(12)
olive flounder (*Paralichthys olivaceus)*	NPY, PYYa, PYYb		Growth hormone expression, food intake, growth	(204, 213, 214)
Medaka (*Oryzias latipes*)	NPYa, NPYb, PYYa	Y2, Y2-2, Y4, Y7, Y8a, Y8b	?	(215, 216)
Tiger puffer (*Takifugu rubripes*)	NPYa, NPYb, PYYa, PYYb	Y2, Y4, Y7, Y8a, Y8b	Food intake	(215, 217, 218)
Spotted green pufferfish (*Tetraodon nigroviridis*)	NPYa, NPYb, PYYa, PYYb	Y2, Y4, Y7, Y8a, Y8b	?	(218, 219)
Nile tilapia (*Oreochromis niloticus*)	NPYa, NPYb, PYYa, PYYb	Y2, Y2-2, Y4, Y7, Y8a, Y8b	Food intake	(220)
African cichlid fish (*Astatotilapia burtoni*)	NPY	Y8a, Y8b	Food intake, reproduction	(221)
Cunner (*Tautogolabrus adspersus*)	NPY		Food intake	(222)
Orange-spotted grouper (*Epinephelus coioides*)	NPY	Y2, Y8b	Food intake, development	(182, 223, 224)
Cobia (*Rachycentron canadum*)	NPY		Food intake	(225)
Snakeskin gourami (*Trichogaster pectoralis*)	NPY		Food intake	(226)
Three-spined stickleback (*Gasterosteus aculeatus*)	NPYa, NPYb, PYYa, PYYb		?	(215)
Siberian sturgeon (*Acipenser baerii*)	NPY	Y1, Y4, Y5, Y6	Food intake	(189, 227, 228)

?, unknown yet.

NPY family peptides exert their physiological functions by binding to their receptors, which are referred to as NPY family receptors. NPY family receptors are G protein-coupled receptors that consist of multiple members. In teleost, there are seven types of NPY receptors, which belong to Y1 family, Y1, Y4, Y8a and Y8b, and Y2 family, Y2, Y2-2, Y7, respectively (187). The *y1* gene is only found in the zebrafish genome (189), speculating that most teleost lost Y1. Studies have revealed that Y4, Y8a, Y8b, Y2 and Y7 are all highly expressed in the brains (190, 218), eyes, heart, kidney, liver, intestine and gonads (182, 220). Research on the distribution of teleost NPY family receptors in brain regions, especially in various sub-regions other than the hypothalamus, remains to be studied.

#### 3.1.2 NPY family in the regulation of fish feeding and growth

The binding activity of NPY family receptors to ligands has been studied extensively in mammals, and a variety of receptor-specific agonists and antagonists have been screened, providing a basis for studying receptor-mediated physiological functions (231). However, knowledge about the ligand-binding activity of NPY family receptors is rare in teleost fish, and mainly focused on zebrafish and rainbow trout (*Oncorhynchus mykiss*) (190, 207). In zebrafish, Y4, Y8a, Y8b, and Y2-2 all have similar binding activities with NPY, PYYa, and PYYb (190, 191). While the affinity of Y2 with PYYb is about 50 times higher than that of PYYa and the affinity of Y7 with PYYa is about three times lower than that of NPY and PYYb (190, 191). In addition, unlike mammalian Y2, the two Y2 family receptors in rainbow trout, Y2 and Y7, are both sensitive to NPY with N-terminal deletion and have minimal binding capacity to NPY (13–24, 27–29, 31, 53, 65, 79, 80, 83–85) and NPY (18–24, 27–29, 31, 53, 65, 79, 80, 83–85), and neither binds to the mammalian Y2-specific antagonist BIIE0246 (207).

Research on the role of NPY in the regulation of bony fish feeding is mainly focused on NPYa. In the available literature, the orexigenic effects of NPY can be triggered by injection, feeding and immersion. Intracerebroventricular (*icv*) injection of NPYa in grass carp (195) and zebrafish (192), as well as intraperitoneal (*ip*) injection of NPYa in red tilapia (*Oreochromis* sp.) (232) and orange-spotted grouper (*Epinephelus coioides*) (223) stimulate food intake. Oral administration of synthetic NPYa significantly increased the growth rate, body weight gain and feed conversion ratio of orange-spotted grouper after feeding for 50 days (223), as well as in tilapia (233). In addition, immersing catfish (*Clarias gariepinus*) fry with recombinant NPYa can also increase their growth rate (234). There are relatively few reports on the regulation of feeding by PYY in teleost fish. In goldfish, PYYa *ip* (10 ng/g BW) or PYYa *icv* (5 ng/g BW) significantly reduced the food intake (197). Also, in Siberian sturgeon (*Acipenser baerii*), PYYa *ip* (10, 100 and 200 ng/g BW) significantly decreased food intake, while low dose of PYYa (1 ng/g BW) has no significant effect on feeding (227). Moreover, in tilapia, PYYa (50 ng/g BW) has no effect on food intake but PYYb *icv* (50 ng/g BW) significantly decreased feeding (220).

The role of NPY receptors in mediating NPY regulation in bony fish feeding is mainly verified based on the agonists and antagonists of mammalian NPY receptors. In goldfish and zebrafish, Y1 antagonist BIBP3226 *icv* inhibits the orexigenic effects of NPY (16, 192). ICV injection of Y1 agonist (Leu31, Pro34)-NPY and Y5 agonist (D-32Trp)-NPY promoted the food intake in goldfish, but neither the Y2 agonist NPY (2–24, 27–29, 31, 53, 65, 79, 80, 83–85) nor the (Pro13, Tyr36)-NPY (13–24, 27–29, 31, 53, 65, 79, 80, 83–85) has significant effect on feeding behaviors, suggesting that NPY receptors similar to mammalian Y1 and Y5 may be involved in feeding regulation in teleost (235, 236). On the other hand, in rainbow trout, ICV injection of both the Y1 agonist (Leu31, Pro34)-NPY and the Y2 agonist NPY (3–24, 27–29, 31, 53, 65, 79, 80, 83–85) stimulated food intake (208). Recently, it is reported that ICV injection of Y1 agonist (Leu31, Pro34)-NPY and Y2 agonist NPY (13–24, 27–29, 31, 53, 65, 79, 80, 83–85) both increased the appetite of Siberian sturgeon within 30 min (228). Unexpectedly, (Leu31, Pro34)-NPY does not specifically recognize NPY receptors in teleost (237), while BIBP3226 has an extremely low binding capacity with Y4, Y8a and Y8b in zebrafish (237). In addition, NPY (3–24, 27–29, 31, 53, 65, 79, 80, 83–85) and (13–24, 27–29, 31, 53, 65, 79, 80, 83–85) cannot specifically recognize Y2 of teleost and have poor binding capacity to Y2 in both rainbow trout and zebrafish (207) but have good binding capacity to Y4 of zebrafish (237). Therefore, the above studies can only infer the potential role of NPY receptors in teleost feeding by the performance of these agonists. The specific types of NPY family receptors involved in the regulation of teleost feeding remain to be studied.

In recent years, researchers have begun to use powerful gene editing techniques to study the function of NPY and its receptors. It has been reported that specific knockout of NPY in AGRP neurons reduced locomotion and energy expenditure and accelerated feeding and respiratory quotient in mice, and the study suggested that NPY originating from AGRP neurons is critical for initiating and sustaining feeding (103). Targeted disruption of the Npy1r gene in limbic areas revealed that limbic NPY-Y1R system was involved in energy balance and emotional behavior, and selective inactivation of limbic Npy1r gene increased susceptibility to diet-induced obesity in male mice (230). Another study showed that mice with β-cell specific ablation of the Y1 exhibit significantly upregulated serum insulin levels associated with increased body weight and adiposity (110). Zebrafish with global knockout of NPY have been found to exhibit some anxiety-like behaviors, indicating an important role of NPY in the regulation of emotional behaviors (95, 96).

### 3.2 Somatostatin and its receptors

Somatostatin (SS or SST), also known as somatotropin release-inhibiting factor (SRIF), is a cyclic polypeptide consisting of 14 amino acids. It was originally isolated from the hypothalamus of sheep and was named for its ability to inhibit the secretion of pituitary GH (18). SS is conservative and widespread in vertebrates, from fish to mammals, and it is also a multifunctional tetradecapeptide involved in a variety of physiological processes such as growth, development, metabolism, reproduction and immunity mediated by specific G protein-coupled receptors (238). Like other polypeptide hormones, SS is a mature peptide produced by tissue-specific enzymatic processing of its precursor protein (preprosomatostatin, PSS). In teleost, SS peptides were firstly identified from anglerfish (*Lophius litulon*) and spotted catfish (*Anarrhichas minor*), and subsequently their cDNAs were cloned (239, 240). Since then, multiple forms of SS peptides and their PSS cDNAs have been identified in different fish species (**Table 4** for detail). Up to date, PSS is a recognized polygene family consisting of six homologous genes, namely *pss1*, *pss2*, *pss3*, *pss4*, *pss5*, and *pss6* (270, 271). A comparative genomic approach reveals that *pss1*, *pss2*, and *pss5* emerged from the second round of genome duplication early in vertebrate evolution; *pss4* was a homolog of *pss1* from the third round of genome doubling (3R) in most teleost; *pss3* and *pss6* arised from tandem duplication of *pss1* and *pss2*, respectively (270, 271).

**Table 4:** Somatostatin and its receptors have been identified in fish.

Species	Prepro-somatostatin	Somatostatin(SS)	Receptors(SSTR)	Physiological functions	Major references
Orange-spotted grouper (*Epinephelus coioides*)	PSSI,PSSII, PSSIII	SS14, SS28	SSTR1;SSTR2; SSTR3;SSTR5	Growth regulation	(241–243)
Goldfish (*Carasius auratus*)	PSSI,PSSII, PSSIII	SS14, SS26, SS28	SSTR1A, B;SSTR2; SSTR3A, B;SSTR5A, B, C	Growth regulation	(244–246)
Anglerfish (*Lophius piscatorius*)	PSSI, PSSII	SS14, SS28	?	Growth regulation	(239, 247)
Rainbow trout (*Oncorhynchus mykiss*)	PSSII, PSSII’	SS14, SS25	SSTR1A, B;SSTR2	Growth regulation, carbohydrate metabolism, food intake	(248, 249)
Zebrafish (*Danio rerio*)	PSSI, PSSII, PSSIII	SS14, SS22	*SSTR1A, B;*SSTR2A, B;*SSTR3;*SSTR5	Growth regulation	(153, 250)
Carp (*Cyprinus carpio*)	PSSIa,b*PSSIIa,b*PSSIIIa,b	SS14,	*SSTR1;*SSTR2; *SSTR3;*SSTR5	Growth regulation	(251)
Catfish (*Ictalurus punctata*)	PSSI, PSSII	SS14, SS22	*SSTR1A, B;*SSTR2A, B;*SSTR3;*SSTR5	Growth regulation, carbohydrate metabolism	(252, 253)
Cichild fish (*Astatotilapia Haplochromis*)	PSSI	SS14	SSTR2; SSTR3	Growth regulation, social behavior	(254–256)
Topmouth culter (*Erythroculter ilishaeformis*)		SS14	SSTR6; SSTR7	Selenium metabolism	(257, 258)
*Siniperca chuatsi*	PSSI, PSSII, PSSIII	SS14	SSTR2; SSTR3	*Digestion regulation, *reproductive regulation	(259)
*Coris julis*			SSTR2; SSTR5	Neural regulation	(260, 261)
Nile Tilapia (*Oreochromis niloticus*)	#PSSI, PSSII, #PSSIII	SS14, SS28	#SSTR2A、B;#SSTR3A、B; #SSTR5	Growth regulation, social behavior, reproductive regulation	(262–264)
Flounder (*Platichthys flesus*)	PSSI, PSSII	SS14, SS28	*SSTR2; *SSTR5	Osmoregulation	(265)
European eel (*Anguilla anguilla*)	PSSI, PSSII	SS14, SS25	*SSTR1A, B;*SSTR2A, B;*SSTR3;*SSTR5	Growth regulation, osmoregulation	(266, 267)
Sea lamprey (*Petromyzon marinus*)	PSSa, PSSb, PSSc	SS14, SS34, SS37	*SSTR1;*SSTR4*SSTR5	Growth regulation, neural Regulation	(268, 269)

*, Sequences or functions are predicted from cDNA. #,WS Li’s lab cloned, unpublished.?, unknown yet

The function of SS is mediated by its seven transmembrane G protein-coupled receptors. Two members of the SSTR family, SSTR1 and SSTR2, were characterized from mice and human (272). SSTR3 was first reported in electric fish (*Apteronotus albifrons*) (273). It has been well reported that there are some receptor subtypes of SSTR in teleost. (For detail, **Table 4**). For example, the full-length cDNA sequences of four SS receptors (SSTR1, SSTR2, SSTR3 and SSTR5) were cloned from orange-spotted grouper (183). There were five SS receptors, SSTR2a (*MW848786*), SSTR2b (*MW848787*), SSTR3a (*MW848788*), SSTR3b (*MW848789*) and SSTR5 (*MW848790*), in Nile tilapia, which were highly homologous to the corresponding receptors of other known vertebrates.

Although SS is involved in a variety of physiological processes, its vital function is to inhibit the basal and induced GH secretion in the pituitary. Related research on SS function in teleost fish has mainly focused on the regulation of pituitary GH secretion. *In vitro* and *in vivo* studies in various teleost, including goldfish (244), tilapia (262), catfish (252) and rainbow trout (241), SS has been demonstrated to inhibit GH secretion. In goldfish, three precursor genes encoding SS (*pss-Ⅰ*, *pss-Ⅱ* and *pss-III*) showed different distribution patterns in regions of the hypothalamus associated with feeding and olfaction, hinting that SS may control food intake in fish (274). Treatment with SS14 has been observed to reduce food conversion, plasma IGF-I and INS levels, and further to causes growth retardation in rainbow trout (275, 276). In addition, SS may also indirectly influence feeding behavior. Reported as a major inhibitor of GH secretion, SS was ultimately found to increase food consumption due to its feeding-prolonging effect (277), which resulted from the direct function of SS to inhibit GH secretion (278). RNA-seq analysis of feeding and non-feeding anadromous fish (*Coilia nasus*) suggests that SS may be involved in the regulation of food intake and metabolic state (65).

Regarding the mechanism of SS on inhibiting growth, it has been revealed that SS inhibited GH secretion with the involvement of cAMP, protein kinase C (PKC), and Ca^2+^ in goldfish (279). Similarly, blockade using the PLC/IP/PKC pathway reversed SRIF-inhibited of GH release without affecting GHRH-stimulated of GH release in grouper (252). These studies suggest that SS inhibited GH secretion through the PKC pathway (280). However, in halibut (*Psetta maxima*), the PKC agonist TPA had no effect on GH secretion, and TPA promoted GH secretion only in the presence of SS (281). SS was found to have no effect on GH secretion induced by PKC agonists in chickens (282), and SS did not inhibit TPA-induced GH secretion in bovine pituitary cells (283). These results show a distinct species specific role for the PKC pathway, which is or is not involved in the process by which SS inhibits GH secretion. Generally, SS is considered as an inhibitor of GH secretion, however, studies in pigs and baboons have found that low concentrations of SS can also promote GH secretion (284, 285). In addition, studies in pigs found that the phospholipase C (PLC) inhibitor U73122 did not block SS-induced GH secretion (286). Similarly, in baboons, both U73122 and the PKC inhibitor Go6983 failed to block SS-induced GH secretion (284).

Studies in mammals have confirmed that activation of SSTRs inhibits cAMP levels and that the AC/cAMP/PKA pathway is involved in the physiological function of SS (287, 288). In goldfish, SS blocked GH secretion induced by the AC agonist forskolin and the cAMP analogue 8-Br-cAMP, suggesting that SS may inhibit GH secretion through the cAMP pathway (279). However, in halibut forskolin had no effect on GH secretion, and forskolin promoted GH secretion only in the presence of SS (281). In contrast, in pigs, SS increased cAMP levels and the AC/cAMP pathway mediated SS-induced GH secretion (286). Identically, SS promoted GH secretion *via* the AC/cAMP/PKA pathway in baboons (284). Taken together, the role of SS in regulating GH secretion differs among species, the AC/cAMP/PKA pathway (238) or the PLC/IP3/PKC pathway (240) is involved in SS-regulated GH secretion.

Nitric oxide (NO) is now recognized as an important signaling molecule involved in the regulation of SS function. NO is produced by NO synthase (NOS) in a reaction that catalyzes the conversion of arginine to citrulline (289). Typically, NO activates downstream ornithine cyclase (OC), which catalyzes the production of cGMP from GTP (290). NO promotes basal GH secretion in rat (291), pig (292), dog (293), and human (294). Conversely, NO was found to inhibit basal GH secretion in the mouse (295) and in the murine pituitary tumor cell line GH3 (296). In addition, it has also been reported that NO is not involved in basal GH secretion (297, 298). Interestingly, in human pituitary tumor cells, depending on its concentration, NO either promotes or inhibits basal GH secretion (299). There is abundant evidence confirming the involvement of NO in basal GH secretion, but its precise role and its mechanism remain highly controversial.

### 3.3 Interaction between neuropeptides

The regulation of feeding is a very complex process involving the modulation of various neuropeptides in the central nervous system. These neuropeptides are inextricably linked to each other. NPY, SS, orexin, etc. have been shown to be involved in feeding regulation, and whether there is a regulatory relationship between these neuropeptides has been a matter of interest to researchers. It is interesting to note that some functionally similar neuropeptides co-localize in brain regions. It has been reported that SS co-localizes with NPY in the mammalian amygdala (300). A double label *in situ* hybridization histochemistry has shown that NPY and SSTR1 are co-expressed in the arcuate nucleus (301). It has been shown that treatment of NPY and SS in the prepyramidal cortex alters the intake of essential amino acid deficient diets in rats, and the cytoarchitecture suggests that NPY and SS-containing neurons in the prepyramidal cortex may be synaptically or polysynaptically associated with local circuit. It is suggested a possible association between these two neuropeptides during electrical activity, olfactory information regulation and neural perception of essential amino acids (302, 303). Pharmacological evidence suggested a role for NPY in the feeding regulation of SS. An agonist of SS, ODT8-SST *icv* injection increased food intake in non-fasted rats during both the light and dark phases, whereas both the SSTR2 antagonist and the Y1 antagonist BIBP-3226 blocked the orexigenic effect of ODT8-SST (304), suggesting that ODT8-SST enhances feeding behavior through SSTR2 involving the activation of Y1. Similar to NPY, orexin is also involved in the feeding regulation of SS. It was found that the antagonist of the orexin receptor, SB-334867, completely blocked the orexigenic phenomenon induced by ODT8-SST (305). In contrast, pretreatment with the SSTR2 antagonist S-406-028 did not affect the orexigenic effect of orexin, indicating that the orexin system is part of the neural circuitry in the brain regions of the orexigenic response induced by *icv* injection of ODT8-SST, and that the orexigenic effect of orexin is independent of SSTR2 (305). In neonatal chicken, SS *icv* injection was shown to significantly increase food intake, and the Y1 antagonist B5063 and Y5 antagonist SML0891 alone showed a dose-dependent decrease in feeding, while the Y2 antagonist SF22 treatment showed a dose-dependent increase (306). The researchers found that co-injection of SS and Y1 antagonist B5063 significantly reduced food intake, however, Y2 antagonist SF22 and Y5 antagonist SML0891 had no significant effect on the feeding behavior induced by SS, these results suggest that the NPY-Y1 system is involved in the feeding regulation of SS (306).

There is no direct evidence in fish to suggest that there is a correlation between NPY and SS in feeding regulation. However, some indirect evidence in fish indicate that NPY and SS are interrelated. In green molly (*Poecilia latipinna*), SS-immunopositive neurons were also found to express NPY (307). In goldfish, NPY and SS were found to be highly co-localized in neurons in the ventrolateral telencephalon (VI), the entopenduncular nucleus (NE) and, to a lesser extent, the dorsocentral nucleus (Dc) of the telencephalon, and in the brainstem (308). Incubation of Spotted Sea Bass (*Lateolabrax maculatus*) brain cells with neuropeptide FF (NPFF) significantly upregulated *orexin* and *npy* mRNA levels and significantly downregulated *ss* mRNA levels (309). Antidepressant Sertraline (SER) stress in juvenile yellow catfish (*Tachysurus fulvidraco*) was found to affect feeding and growth by regulating transcript levels of *npy*, *ss* and *gh* (30).

## 4 Other neuropeptides related to fish feeding and growth

Feeding and growth is regulated not only by the influence from complicated external environment, but also the integrate regulations at the intrinsic molecular level. In addition to the above-mentioned NPY family and SS family, there are other neuropeptides involved in fish feeding and growth regulation.

Cholecystokinin (CCK) is a brain-gut peptide secreted by both small intestinal mucosal I cell and central nervous cells. There are some forms of CCK precursor mRNA found in fish, which can be divided into two clusters by performing evolutionary tree analysis, so called CCK-1 and CCK-2 (310). CCK is an important peripheral inhibitor of feeding, and it may also be one of the factors that integrally regulate feeding and growth hormone release (311–313). CCK inhibited gastric emptying and promoted gallbladder contraction in rainbow trout and other salmonids (311, 312). The mRNA expression of *cck* was reduced during starvation and significantly increased after feeding in Atlantic salmon (*Salmo salar*) (311), Japanese flounder (314), winter skate (*Raja ocellata*) (315) and yellowtail (230). The mRNA expression of CCK in the pyloric caecum of goldfish and yellowtail was also elevated after feeding, and further research revealed that intraventricular and IP injection of CCK into goldfish could inhibit food intake (313). Oral ingestion of CCK reduced dietary intake in European sea bass (*Dicentrarchus labrax*), whereas CCK antagonists administered enhanced dietary intake in sea bass and rainbow trout (316). IP injections of CCK8 with 100 and 200 ng/g BW in Siberian sturgeon (*Acipenser baerii Brandt*) showed that CCK8 inhibited ingestion from 0-1 h and cumulative ingestion at 3 h. Chronic injections of 100 and 200 ng/g BW CCK8 at 7 d resulted in a significant reduction in daily and cumulative ingestion (317). These results suggest that CCK also acts as a satiety signal in fish and is one of the factors that integrally regulate fish food intake.

The corticotropin-releasing factor (CRF) system consists of four homologous lineages, two major receptors (CRF-R1 and CRF-R2) and a binding protein CRF-BP. The homologous gene lineages are corticotropin-releasing factor (CRF), urotensin I (UI)/sauvagine (SVG)/urocortin 1 (UCN1), urocortin 2 (UCN2) and urocortin 3 (UCN3), with UI, SVG and UCN1 as homologs (20, 318). It has been found that the CRF system is associated with fish feeding. The mRNA expression of *crf* was significantly down-regulated by fasting goldfish for 7 days and up-regulated by overfeeding (319). Pre- and post-prandial, fasting and refeeding researches on Dabry**’**s sturgeon (*Acipenser dabryanus Dumeril*) showed that *crf* mRNA elevated significantly at 1 h and 3 h after feeding, *crf* and *crf* -Rs transcripts significantly reduced at 10 d of fasting but elevated at day 10 of refeeding (320). ICV of CRF or UI treatment were found to reduce the feeding quantity in rainbow trout (321). On the other hand, the mRNA expression of *crf* decreased significantly when *Schizothorax prenanti* fasting for 7 days but increased significantly after re-feeding (322). IP injection of different doses of UCN3 into Siberian sturgeon showed a reduction in feeding from 0 to 6 hours, and with 7 days of injection, the cumulative amount of feeding reduced significantly compared to the control group (323). The above findings suggest that the CRF system may act as a feeding inhibitor in fish.

Two orexins (also known as hypothalamus), orexin-A (OXA) and -B (OXB), were first characterized from rat hypothalamus almost simultaneously by two teams (324, 325). Subsequently, orexin cDNA sequences have also been cloned successively from, zebrafish (326), Atlantic Cod (*Gadus morhua*) (327) and Winter Flounder (*Pleuronectes americanus*) (328). Fish orexin neurons concentrate in the hypothalamus and extend to other brain regions such as the ventral medial hypothalamus and dorsal mesencephalon (329). In zebrafish and goldfish, prolonged starvation significantly upregulated brain orexin mRNA expression (330). Goldfish ICV injections of either OXA or OXB can dose-dependently promote feeding, and the effect of OXA is stronger than that of OXB (17). IP injection of OXA in *Thalassoma pavo* also significantly stimulated feeding and locomotion (331). In Atlantic cod, orexin mRNA expression levels in the hypothalamus were significantly higher during ingestion than before and after ingestion (327). Orexin also interacts with other appetite-regulating peptides to regulate feeding. Blocking the orexin receptor pathway would decrease the NPY-induced pro-appetitive effect (22), in the same way, blocking the NPY receptor signaling pathway reduced the orexin-triggered pro-feeding effect (332). It suggests that orexin synergizes with NPY to promote feeding in fish.

POMC is a common precursor of adrenocorticotropic hormone (ATCH), melanocyte-stimulating hormones (including γ-MSHs, α-MSHs, β-MSHs, and δ-MSHs), β-endorphin (β-END) (19). POMC is mainly expressed in the pituitary gland and, together with its derived peptides, i.e., ATCH, MSHs and MSHs receptors such as MC4R, forms the melanocortin system, which is involved in food intake and energy balance (333). When POMC neurons receive stimulation, they release MSHs, which act with melanocortin receptors in the hypothalamus and other brain regions to reduce food intake and increase basal metabolic rate (334). In grass carp feeding experiment, feeding and weight gain rates increased significantly with enhancing levels of the diets, whereas restricted diets significantly reduced the transcript levels of hypothalamic POMC (335). Overfeeding of zebrafish larvae reduced POMC levels and declined activation of MC4R (336). Short-term fasting of snakeskin gourami (*Trichopodus pectoralis*) resulted in the lowest levels of POMC mRNAs in the telencephalon and mesencephalon as well as the pituitary gland at 12 h of fasting, while POMC transcripts reached their lowest point at 6 h of fasting (337). In addition, POMC as feeding-inhibiting neuropeptide in fish, displays a potential antagonistic relationship with other neuropeptides such as AgRP (Agouti Related protein) and NPY. Results from snakeskin gourami indicated that a decrease in MC4R expression was observed 1 h before the last meal of the day, while no such decrease was shown 1 h before the first meal of the day. Postprandial NPY expression decreases, with peak NPY expression occurring 1 hour before the first meal of the day (226). It has been reported that a unique mouse model (Pomc ^tm1Kgm^) which was unable to generate desacetyl-α-MSH and α-MSH from ACTH _1-39_ developed the characteristic melanocortin obesity phenotype, and desacetyl-α-MSH and α-MSH were found to regulate body weight and energy balance in mouse (213). Risperidone treatment was found to cause hyperphagia and induce weight gain, and transcriptomic analyses in the hypothalamus of risperidone-fed mice revealed that risperidone treatment reduced the expression of *mc4r*, and MC4R-specific agonist experiments indicated that the atypical antipsychotic risperidone targets hypothalamic MC4R to cause weight gain (338).

## 5 Summary and outlook

Food intake and growth are two physiological processes that are directly related and affect each other. NPY is an important orexigenic factor in the hypothalamus and has been shown to increase appetite, promote GH secretion and increase growth rate in fish (219, 223). SS is widely known for inhibiting the growth of animals earlier, but later studies show that SS also takes part in the regulation of feeding in fish (65, 274). Similar to SS, neuropeptides initially identified for other functions are later shown to be involved in feeding regulation in fish, such as CRF, which are originally known to regulate reproduction and promote corticotropin secretion, respectively (339).

Currently, studies on the regulation of feeding mechanisms in fish are still at infancy, and most of them are limited to the analysis of neuropeptides and receptors at the mRNA level, including the molecular cloning and tissue distribution characterization of neuropeptides and their receptors, but lacking the exploration of its feeding regulation mechanism. Moreover, the analysis of the tissue distribution of neuropeptides and receptors is relatively shallow. In the brain area, there are many feeding-related neuropeptides and receptors. However, most studies do not accurately delineate brain regions, but use the entire brain or the entire hypothalamus for tissue distribution analysis.

Although the basic mechanisms of food intake regulation in mammals and fish are relatively conserved, there are still huge physiological differences. In addition, teleost have also undergone the 3R (340). Compared with other groups, fish have more types of neuropeptides and receptors related to feeding. What’s more complicated is that multiple protein subtypes may have different physiological functions, which makes the study of their feeding regulation mechanism more challenging. There are still many gaps to be filled in understanding the regulation mechanism of fish ingestion.

Advances in omics and gene editing technologies have allowed us to study the function of neuropeptides and their receptors in greater depth. At present, the most used omics technology in the study of neuropeptide and its receptor function is transcriptomic technology, which mainly focuses on mammals. It is expected that researchers can focus more on non-mammals and use proteome or metabolome to explore the field of neuropeptides from multiple dimensions. Currently, both systemic knockout and conditional knockout transgenic animals are available for the study of neuropeptides and their receptors. Conditional knockout can be more targeted to study the function of neuropeptides and their receptors in a certain tissue or type of cells, but unfortunately, conditional knockout has only been reported in mice applying cre/loxp system. In the future, researchers may try to apply CRISP/Cas9 to perform tissue/cell-specific knockout of neuropeptides or their receptors in non-mammalian animals.

## Author contributions

XY and HY contributed to the literature review and writing of the manuscript. WL contributed to review and editing of the manuscript. All authors contributed to the article and approved the submitted version.

## Funding

This work was supported by the National Key R&D Program of China 2018YFD0900101, China Agriculture Research System (CARS-46), and National Science Foundation of China (32072968) to WL.

## Conflict of interest

The authors declare that the research was conducted in the absence of any commercial or financial relationships that could be construed as a potential conflict of interest.

## Publisher’s note

All claims expressed in this article are solely those of the authors and do not necessarily represent those of their affiliated organizations, or those of the publisher, the editors and the reviewers. Any product that may be evaluated in this article, or claim that may be made by its manufacturer, is not guaranteed or endorsed by the publisher.

## Abbreviations

3R, third round of genome doubling; CCK, cholecystokinin; CRF, corticotropin-releasing factor; GH, growth hormone; GHR1, growth hormone receptor 1; GHRH, growth hormone-releasing hormone; ICV, intracerebroventricular; IP, intraperitoneal; NPY, neuropeptide Y; POMC, proopiomelanocortin; PP, pancreatic polypeptide; PSS, preprosomatostatin; PYY, peptide YY; SS, somatostatin; SSTRs, somatostatin receptors; SVG, sauvagine; UCN1, urocortin 1; UCN2, urocortin 2; UCN3, urocortin 3; UI, urotensin I.
